# The Effects of the Socialization of Physical Education Teachers on Their Modes of Interaction With Students in Tunisian Schools

**DOI:** 10.3389/fsoc.2021.747092

**Published:** 2022-02-02

**Authors:** Bechir Nasri, Abdelmotaleb Kadri, Nizar Souissi, Mourad Rouissi

**Affiliations:** Department of Sport Sciences, Higher Institute of Sport and Physical Education of Ksar Saïd, University of Manouba, Tunis, Tunisia

**Keywords:** socialization, culture, social integration, interaction, educational practice

## Abstract

This article uses an interactionist perspective to address the socialization of physical education teachers in a teaching setting in Tunisia. The two main objectives of the study are to present the social characteristics of the population studied, draw general conclusions about the social origins of the Tunisian teachers of physical education and sport, and to identify the link between socialization and the pedagogical practice of physical education teachers in Tunisian schools. My colleagues and I collected data through participant observations and in-depth interviews with a representative sample of teachers. The results show that the socio-cultural origin, the type of training and the generational effect of physical education teachers in a teaching setting in Tunisian schools influence pedagogical behavior.

## Introduction

Sociologists describe socialization as the process of acquiring the norms and values of a society, or a particular social group (Darmon, 2006). These values and norms, internalized by individuals during their existence (Darmon, 2010) will determine their behavior (Dubar, 1991), their way of thinking, and acting in society. During primary socialization, the child is particularly easily influenced and the first experiences have a strong hold on him (Durkheim, 1937; Durkheim, 1997).

Our reflection in this approach focuses on the relationship between socialization and the pedagogical practice of physical education teachers in Tunisian schools. We are particularly interested in the relationships between socialization and teachers, their ability to transmit pedagogical knowledge and their modes of interaction with students within the framework of teaching.

To understand the pedagogical practice of this group of teachers, it is necessary to study certain aspects of their social origin (Bourdieu, 1979; Bourdieu and Passeron, 1964), and their cultural capital (Bourdieu and Passeron, 1970; Bourdieu, 1980) but also their training within the institutes. superiors of sport and physical education in Tunisia.

This approach makes it possible to identify the transformations that affect the different dimensions of the teacher’s personality (Parsons and Bales, 1955), and this by putting the theory of socialization into practice in a socio-professional context (Dubar, 1991). Emphasis will be placed on teacher training in Tunisia and in particular on the profile of the PE teacher^1^ at the end of his university career and on his ability to transmit their pedagogical knowledge.

Being a teacher is the opportunity to renew yourself every day, to adapt to the profile of each student, to allow them to develop their potential, and transmit their values. (Parlebas, 1986; Lautrey, 1995; Feyfant, 2008), and ultimately, to be an actor in an evolving education system.

The question we are trying to answer: How do the different forms of socialization of PE teachers influence their conceptions and their modes of interaction with students within the framework of teaching? Our issue emphasizes the role that the socialization of PE teachers can play on their teaching practice with their students in the Tunisian school.

What is the role of socialization on the teaching practices of the physical education teacher and the modes of interaction with the pupils?

What is the influence of the context in which the teacher evolves: how does the context influence the teaching practice between the teacher and the pupils?

Socialization, in particular social origin, academic capital, and training influence (Draelants and Ballatore, 2014) the pedagogical practice of PE teachers and their interactions with students. Finally, depending on the establishment and the students, teachers do not have the same practices. The institutional environment, the socio-professional environment, would have an influence on the behavior of teachers, and their mode of interaction with students.

### The Analytical Framework

From pedagogical practice, verbal and motor intervention, which is established between the PE teacher, and the student in a teaching and learning situation during a PE session (Arnaud, 1992). We will study the socialization of these groups of teachers, in order to know the pedagogical and behavioral aspects towards the pupils within the framework of teaching and to see the relation between the socialization and the pedagogical practice of the teachers.

More precisely, we will be interested in the influence of the socialization of teachers on their conceptions and their modes of interaction (Cunff and Hugon, 2011) with students in the context of education, and on their ability to transmit learning outcomes.

We position the study of socialization in an interactional field, from an interactionist perspective (Garfinkel, 1967), to focus on teacher training in Tunisia and in particular, on the educational and social profile of the teacher at the end of his career. university, and on its ability to transmit its pedagogical knowledge.

According to this approach, an individual’s personality is never given once and for all. (Montousse and Renurd, 1999) it is reinforced or modified by each of the relationships between the individual and the others (Montousse and Renurd, 1999). This analysis will be based on the theoretical tendencies of Erving Goffman, in which the norms and the social roles are not data which are imposed on the individuals (Goffman, 1974; Bulmer, 1962), they are constructions which appear, or are transformed in the core of multiple interactions (Goffman, 1974; Becker, 2004).

To assess PE teachers we will refer to the notion of competence, a notion widely used in the fields of education, training, guidance and human resources.

In psychology, competence is an identity term which covers both capacities and aptitudes. It is defined as a positive characteristic of an individual testifying to their ability to accomplish certain tasks (Guichard and Huteau, 2007; Huteau, 2018).

Specific competence is the set of individual characteristics that allow an individual to master a given situation through effective activity. These characteristics form a dynamic system, the result of which is precisely competence.

The competence calls upon two concepts: Aptitudes considered as natural, relatively complex dispositions, revealed and confronted by the environment and social learning. They make it possible to approach a task with ease (Claparède, 1953) and constitute an asset for acquiring a capacity. The capacity being the set of knowledge learned. When we combine capacity and aptitude, we have competence; the competence then being the synthesis of the initial data and the acquisitions.

## Methodology

As part of our study and in order to find answers to the hypotheses formulated previously, we opted for a qualitative approach. Thus, in order to obtain detailed information on the influence of socialization on teaching practice and on teacher behavior, two data collection methods were employed.

First of all, we havecarried out a year of observation of the teaching practices of PE teachers. Indeed,we haveAttended thirty-five course sessions in two high schools in Tunis, a pilot high school and another sportsman. The sessions observed, lasting 2 hours each, took place in the morning and afternoon. Some were considered as training sessions reserved for development, others as learning sessions intended for the assessment of prior learning. As a PE teacher, we have succeeded in integrating us perfectly into the two schools in question. So, we have had the opportunity to observe teachers’ interactions with their students throughout the 2019–2020 school year.

In our observation, we will adopt a grid, which consists of two variables, first, verbal communication such as, speaking language, tone of sound, terms, and speech produced in the classroom. Then, the non-verbal communications which are, the touch, the facial expression, the look, the physical appearance, and the movement of the teacher.

We also conducted interviews semi-structured individuals with13 teachers: 7 men and 6 women. This maintenance method allowed us to leave a certain margin of freedom for the people questioned and offered them the possibility of expressing themselves at their ease. The 13 interviews, each lasting approximately 30 min, took place face to face, between October 2019 and March 2020. Teachers were selected on the basis of several criteria such as age, grade, gender, and seniority. The selection of the sample was motivated by a reasoned choice and therefore in a non-probabilistic manner.

Opting for a pilot public school and a public sports school allowed me to enrich the phenomenon of socialization. Likewise, the choice of a school audience and a varied teaching body made it possible to offer a more diversified and broader panorama of reality, thus offering a global vision of the problem dealt with.

## The Results

### The Socio-Cultural Origin of the Respondents

Table 1 presents the socio-cultural and professional characteristics of the population studied. In order to carry out our work, we have designed an observation grid (qualitative data) which is based on the study of verbal interaction, the type of speech produced during PE sessions and the type of feedback obtained. We chose three variables, namely the lesson demonstration, the pedagogical interactions with the students and the vocabulary used. We believe that these variables are essential for direct field observation, because they can encompass all possible analysis parameters. The direct observations of most teachers in the field, show that the teaching of physical education and the interaction between students and teachers revolve around two pedagogical aspects.

**TABLE 1:** Sociocultural characteristics of the study population.

Rank	Sex	Age	Teaching practice	Cultural and social interest	Sporting career		Upbringing	Native origin
					Sporting course	Professionnal career		
1	Man	33	5 years of seniority	No cultural interest	Athlete, 5 years old basketball	Work in a private gym	Strict	Urban
2	Man	36	8 years seniority	No cultural interest	Former player (windsurfing)	No activity	Liberal	Urban
3	Man	41	16 years old	No cultural interest	Former fencing player	No activity	Strict	Rural area
4	Man	57	35 years	No cultural interest	National team coach	Former coach abroad	Authoritarian and strict	Rural area
5	Man	49	24 years	No cultural interest	Former table tennis player	No activity	Strict	Urban
6	Man	55	28 years	Theater	Former athletics player	National team coach	Conservative	Urban
7	Man	50	29 years	No cultural interest	Former athletics player	Work in a private gym	Conservative	Rural area
8	Women	38	15 years	No cultural interest	Former Hand Ball player	She teaches in a private high school	Conservative	Rural area
9	Women	44	17 years	No cultural interest	Former volleyball player	No activity	Liberal	Urban
10	Women	50	30 years	No cultural interest	Former judo player	No activity	Conservative	Urban
11	Women	36	10 years	No cultural interest	Former athletics player	No activity	moderate	Urban
12	Women	45	20 years	No cultural interest	Former athletics player	Work in a private gym	Liberal	Urban
13	Women	51	31 years	No cultural interest	Former handball player	No activity	Conservative	Rural area

Table 2 presents the gestures, touches, facial expressions, looks, body postures, teachers’ movements, and sound variations during pedagogical interactions with their students. If the teacher had used the visual, tactical and symbolic channel several times, the rating “very frequent” was assigned to him. On the other hand, if he had only used one channel, his rating was qualified as “quite frequent”. Indeed, most teachers are touched their students infrequently, but a minority made little use of this communication. Then we observed that a single teacher who demonstrates his teaching contents with a facial expression visible on the face, when communicating, the expression on his face shows his pleasure, and while the leftovers of the professors are presented his lessons with a weak expressive facial contact. The looks and physical appearance and eye contact of the observed teachers are more frequent throughout the session. Finally, young teachers are moved faster in space more than older ones.

**TABLE 2:** Teachers’ non-verbal gestures.

Non-verbal communication	PE teachers												
	E1	E2	E3	E4	E5	E6	E7	E8	E9	E10	E11	E12	E13
The Contact	QF	QF	VF	QF	QF	QF	QF	QF	QF	QF	QF	VF	VF
Facial expression	QF	QF	QF	QF	QF	QF	VF	QF	QF	QF	QF	QF	QF
Eye contact	VF	VF	VF	VF	VF	VF	VF	VF	VF	VF	VF	VF	VF
Physical appearance	VF	VF	VF	VF	VF	VF	VF	VF	VF	VF	VF	VF	VF
Teacher mouvement	VF	VF	VF	QF	VF	QF	VF	VF	VF	VF	VF	VF	QF

QF: quite frequent.

VF: very frequent.

Concerning the observations of PE teachers, interactions were studied according to three categories according to the socio-cultural origin of the teachers. The first group, the largest in number, is made up of teachers living in semi-urban areas and coming from the middle class. It emerges that overall, the pedagogical interactions are distinguished more by the gestural aspect than by the verbal instruction to explain the content of the PE session. Body language is therefore essential in the relationship between learner and teacher. Our direct field observation emphasizes sign language which is based on operative gestures; it is a gestural lexicon common (Dubar, 2010) to PE teachers and their students.

We have distinguished the different gestures present in the interaction; those accompanying the warm-up phase, those of the warm-up phase and finally the sign language used to guide the learners during the other parts of the session. We can deduce from this that the body makes it possible to interact and exchange technical messages, in order to build non-verbal communication in the teacher-student relationship.

The second group, for its part, comprises a small number of PE teachers, living in urban areas, and coming from a cultivated environment. According to our data, these respondents have liberal rules of life and a moderate pace of life. In addition, when they were children, they practiced various cultural activities such as theater. Our observation made it possible to identify a posture, a gesture, a management of space, and a quality of animation very different from those of the other teachers. The difference appears in particular in the facial expressions and in the movement of the hands and forearms which makes their speech more explicit. These non-verbal behaviors make it possible to encourage the pupils and to evaluate the activities carried out during the whole session. Thereby, interactions with learners are numerous and instructions are frequently explained and intended for all students in the class. However, an EP teacher, a fencing specialist and who teaches his specialty at a sports school, and caught our attention. He comes from the middle class but evolves in a cultivated Universe. Son of an engineer and a housewife, passionate about reading, he brilliantly graduated from university. This teacher is distinguished by his calm during class sessions and by the many advice he gives to the boys and girls in his class. Likewise, he devotes a lot of time to evaluation and verbal interaction, using a language that mixes French and the Tunisian dialect (Quitout, 2007).

However, an EP teacher, a fencing specialist and who teaches his specialty at a sports school, caught our attention. He comes from the middle class but evolves in a cultivated Universe. Son of an engineer and a housewife, passionate about reading, he brilliantly graduated from university. This teacher is distinguished by his calm during class sessions and by the many advice he gives to the boys and girls in his class. Likewise, he devotes a lot of time to evaluation and verbal interaction, using a language that mixes French, and the Tunisian dialect. However, an EP teacher, a fencing specialist and who teaches his specialty at a sports school, caught our attention. He comes from the middle class but evolves in a cultivated Universe. Son of an engineer and a housewife, passionate about reading, and he brilliantly graduated from university. This teacher is distinguished by his calm during class sessions and by the many advice he gives to the boys and girls in his class. Likewise, he devotes a lot of time to evaluation and verbal interaction, using a language that mixes French, and the Tunisian dialect (Quitout, 2007). Son of an engineer and a housewife, passionate about reading, he brilliantly graduated from university. This teacher is distinguished by his calm during class sessions and by the many advice he gives to the boys and girls in his class. Likewise, he devotes a lot of time to evaluation and verbal interaction, using a language that mixes French, and the Tunisian dialect (Quitout, 2007). Son of an engineer and a housewife, passionate about reading, he brilliantly graduated from university. This teacher is distinguished by his calm during class sessions and by the many advice he gives to the boys and girls in his class. Likewise, he devotes a lot of time to evaluation and verbal interaction, using a language that mixes French, and the Tunisian dialect (Quitout, 2007).

In addition, there are five teachers, three men and two women, and who come from the middle class of a rural area. According to the data collected, their mothers were part of the non-working population and all performed household chores. In addition, in their childhood, these teachers did not learn social skills or practice cultural, and associative activities. We have noticed that their interactions unconsciously involve certain gestures of self-contact: hands clasped, arms crossed or in pockets (Streeck and Knapp, 1992).

On the linguistic level, the PE teachers surveyed show a low linguistic diversity especially concerning those who do not master the French language. In addition, in all parts of the physical education session, the vocabulary used and the speech produced is not appropriate, and is not drawn from the applied and fundamental sciences of PE, such as physiology of effort, anatomy, psychology, sociology, and science education. Similarly, the use of the spoken language, that is to say the Tunisian dialect (Quitout, 2007) and not in French, predominate throughout the session. However, it seems important to us to remember that the use of a language can result from the expression of a choice determined by a type of socio-educational trajectory.

The data collected show that, the visits and cultural practices carried out by our interviewees, during university studies, is the cinema with frequent outings. In addition, trends in theater, classical music as well as diverse cultural practices are the least common among our teachers. Finally, part of the occupations of students outside of courses oriented towards leisure or sports activities in civilian clubs that can be practiced individually, another important part relates to collective activities.

### The Type of Training

Overall, all of the teachers surveyed have had an important and varied sports career. All of them have distinguished themselves in national level competitions, in particular by obtaining individual titles. Likewise, all the teachers have practiced competitive sports and show a certain interest in team sports such as football, handball, and rugby, which corresponds quite well to sporting tastes in popular circles. Only three teachers (one man and two women) are PE teachers. Three respondents (a woman and two men) are masters and mistresses who have followed a first cycle of university after obtaining the baccalaureate. Most of the respondents are PE teachers and hold a master’s degree.

Given that the body of PE teachers in Tunisia is made up of three professional statuses, namely teachers of the master and teacher type of PE who have not obtained the baccalaureate, masters and mistresses with university degrees (Bac +2) and finally PE teachers (Bac +4), we will therefore establish a distinction one between three types of categories of teaching practices.

Direct field observation of three PE teachers who did not obtain their baccalaureate, a man and two women, shows that respondents do not or very little use educational tools like index cards and journals in their classes. In addition, we noted that the teacher belonging to this first category repeated key words based on rather vague terms and present in the verbalizations of the latter. These keywords can be applied to different teaching-learning situations and not just in PE sessions. We cite as examples the terms: “performance”, “better”, “success”, “safety,” “attention ”,“ learn ”,“ concentrate ”,“ try ”. In addition, the two teachers frequently resort to grading, sanctions and threats to manage the class. Only the male teacher relies more on his physical presence to exercise some authority over his students.

The second category, for its part, is made up of teachers and PE teachers, holders of the baccalaureate. The three oldest teachers belonging to this category presented their lessons without resorting to pedagogical and didactic tools, but they explained the content of the lessons clearly and formulated their instructions in detail. Five other teachers (four women and one man) focused on organizational instruction, particularly during the tests that are part of the “sports baccalaureate” examination such as athletics.

Direct field observation of three PE teachers who did not obtain their baccalaureate, a man and two women, shows that respondents do not or very little use educational tools like index cards, and journals in their classes. In addition, we noted that the teacher belonging to this first category repeated key words based on rather vague terms present in the verbalizations of the latter. These keywords can be applied to different teaching-learning situations and not just in PE sessions. We cite as examples the terms: “performance”, “better”, “success”, “safety,” “attention ”,“ learn ”,“ concentrate ”,“ try ”. In addition, the two teachers frequently resort to grading, sanctions and threats to manage the class. Only the male teacher relies more on his physical presence to exercise some authority over his students.

The second category, for its part, is made up of teachers and PE teachers, holders of the baccalaureate. The three oldest teachers belonging to this category, presented their lessons without resorting to pedagogical and didactic tools, but they explained the content of the lessons clearly, and formulated their instructions in a detailed manner. Five other teachers (four women and one man) focused on organizational instruction, particularly during the tests that are part of the “sports baccalaureate” examination such as athletics.

Most of the youngest respondents are distinguished by a certain quality in the use and preparation of didactic tools and in the rational management of time according to the degrees of complexity of the tasks requested. Five respondents underline that the preparation of educational sheets is very important since it projects an ideal image of the situation that one wishes to create. It is a tool which allows both to forge and to enrich the teacher’s experience. This is why it is one of the essential factors for the success of a teaching situation. Finally, we observed similarities between the respondents of the two categories, that is to say the teachers (holders and not holders of a baccalaureate), in terms of the vocabulary used when explaining the educational content during the session. These similarities consist in the use of unscientific terms and in the use of the spoken language which is neither French nor Arabic but the Tunisian dialect.

Most teachers have stressed that the university represents itself as a place of study, even of intangible service, but they do not see it as a Universe for learning social, and interactive skills.

### The Generational Effect

Five respondents are aged 50 and over (three men and two women) while eight of them are under 50. We can say that most of the respondents have a variety of professional activities: teaching in private high schools and training in private sports halls and civilian clubs. Only one teacher worked abroad in private clubs. On the one hand, the direct observations of the older teachers, in the field, show that the teaching practice of the two teachers is characterized by a fairly long gestural demonstration. Two of the teachers, the one who worked as a national team coach as well as the one who worked abroad (Arab Gulf countries) also use the feedback illustrated by examples drawn from their own experiences. Our last teacher in this category emphasizes only one part namely the body of the session and does not give importance to the other parts of the course such as the handling, the start-up and the conclusion of the session. In general, our respondents spoke a lot about their personal experiences of practice and training. The quality of the presentation is totally devoid of any scientific dimension, which is however essential when it comes to explaining the movements of physical activities.

On the other hand, we noticed a certain homogeneity characterizing the teaching practice of our the youngest respondents. These are four teachers, three men and one woman, who are considered creative and active in their teaching practice and have a taste for innovation, particularly in terms of the presentation of the work requested. In addition, there are three teachers who use the internet, and sports science social media to teach. They sought to enrich the work offered by educational tools. Finally, the last teacher in this category, considered multitasking, would have the habit of doing several things at the same time. He is more understanding when faced with students who seem to be doing something quite different than listening to their lessons.

Overall, the relational aspect is mainly observed among teachers in relation to their students of the sports high school, in addition, the importance of this relation in this institution is also evident. On the other hand, we could see that a simple relation to an educational aspect, which exists between the teachers and the learners at the pilot high school.

Our field observation also shows that there is a big difference in conditions and resources observed in the field, in the pilot school, the equipment and materials are very modest, such as the gymnastics room it is not compliant. to universal standards, with a more or less mediocre athletic track. The inadequacy of the material for the learning activities offered by the teachers helps explain the students’ difficulties. In addition, the problems of lack of sports infrastructure and teaching materials affect the quality of education. While in the sports school, the material resources requested are diverse and more sophisticated, such as indoor rooms, swimming pools, and collective sports grounds.

On the other hand, with regard to the effectiveness of the teacher and his competence in teaching, we see here that all the teachers of the two high schools are committed to the application of the official PE programs. However, the teachers of the sports high school and given the commitment of their students to national and international sports participations since they belong to the sports elite, they focus more on technical skills and also on psychological preparation, for to become more competitive, and also, to achieve better sporting results nationally and globally.

The mark obtained is a form of judgment which encompasses complex and diverse elements. Likewise, it does not, on its own, account for the effectiveness of teaching practices. Since our interviewees affirmed that they adopted, a grading system that takes into account not only the success of the PE exams in the classroom, but also athletic performance for the students of the sports high school, and the will and desire of the students regarding the students of the pilot high school.

## Results Interpretation

As part of teaching in the Tunisian school, the results show that the pedagogical behavior is influenced by the socio-cultural origin, the type of training followed, and the generational effect of the teachers of physical education. Indeed, our first result relates to the socio-cultural origin of the teachers since most of them come from the working class. Teaching behavior seems to be linked to the personal experience of the PE teachers interviewed. The family trajectory, the school experience also seem to have an influence on the pedagogical behaviors employed. In addition, we believe that the interactions of physical education teachers with their students non-consciously reproduce inherited family behaviors.

The teachers of rural origin who had a conservative education, with a socialization based on the family, in a way that the children of this social class, did not have cultural, intellectual and social interests which play a fundamental role in qualification for professional and social success. On the other hand, and according to the teachers’ responses, they pointed out that a set of family values and behaviors that were anchored to them during childhood, such as raising their voice and giving orders, in addition to speaking using signs, and body language is considered forbidden and even bad manners of speaking. Therefore, this education contributed to the formation of this behavior and the teacher was constrained by these standards during teaching practice. Otherwise, primary socialization has deepened in the behavior of teachers in pedagogical relations and interaction with students. While being teachers belonging to an open urban environment, they are supported by many tools in addition to continuous and diversified family support of a social, cultural, intellectual and sporting nature, and which qualifies them to succeed in their work and in their practices social.

In PE teaching, posture has been dealt with in several studies, in particular, devoted to the evaluation of the interaction used by teachers as well as the role played by non-verbal interactions of PE teachers, and with their students at school. The place occupied by sign language, independently of any spoken language, and was also highlighted. The hands are primarily used for gesticulated language, but the face conveys emotions and makes it possible to resolve ambiguities or to clarify the meaning of the words.

We can therefore deduce that the PE teacher should not only plan the structure and the exercises that he will give in his course but, he should also think about the way of transmitting the instructions and the influence of its non-verbal communications on student engagement. According to a professor at the University of California (Merhabian and Wiener, 1967) in understanding a message, words count for only 7%, the voice for 38% and the non-verbal for 55% in communication.

This shows the influence that PE teacher’s non-verbal communications can have on students. According to the results retained, most teachers focus their practice on common gestures, in particular body language and gaze, these items were observed and safe as relevant. These gestural aspects, conducted in the classroom, establish, and guarantee the principle of safety when teaching PE.

However, it should be remembered that gestures have a primordial role in the transmission of teaching content and knowledge at school. In addition, during our research, we verified the place of gestures, their use in the teaching of PE, and their impact when they are used by teachers. In this context, we note their direct relationship with socialization.

One of the specificities of teachers is their ability to transmit pedagogical knowledge and their modes of interaction with students. In the case where a teacher is scientifically competent but he does not have the ability to communicate effectively with the pupils, he is therefore not able to teach satisfactorily; thus, the teaching-learning process has not been fully achieved.

The inability to master the French language is partly due to the teachers’ low cultural and academic capital. This reflects the type of socialization of these people. This hinders good verbal communication and explanation of educational content in the teaching process.

In addition, there is a minority of teachers who are distinguished by perfect body language and verbal communication during teaching practice. They know how to adapt the content taught to the needs of their students. This practice is due to a certain cultural, educational and social capital already anchored through primary socialization, as well as the secondary socialization effects of these teachers, especially those who have practiced cultural activities such as theater.

Our second result concerns the type of training and makes it possible to identify two categories of teachers, with reference to their professional status: teachers of the PE teacher and teacher type who did not obtain the baccalaureate, masters and mistresses with university degrees (Bac +2) and PE teachers (Bac +4). In the first category of teachers, gestures dominate in teaching practice. Indeed, having belonged to national teams of different sports disciplines, these teachers are influenced by the achievements of their sports training. Likewise, the explanations provided during their lessons are not very in-depth, because of their rather limited academic level. In addition, the higher institutes of sport and physical education in Tunisia, placed outside campuses and university residences, so that PE students do not have the chance to establish interactions with students, and students from other fields. A minority of respondents said they had taken part in cultural events within their establishment.

In the second category, that of teachers with a bac +2 diet and teachers with a bac +4 diet, the older teachers do not rigorously apply the scientific bases of sports and movement sciences. This is explained in particular by the long period separating their university training and teaching practice. In other words, professional experience not accompanied by continuing education, seminars, and colloquiums can lead to boring teaching behavior.

Younger teachers, for their part, use feedback, linked to scientific knowledge, and movement sciences. Regarding the language used (the Tunisian dialect) (Quitout, 2007), namely Tunisian Arabic during the interviews, the teachers underlined the fact that most of their teachers did not use the French language during their teaching, in practical tests at the University of Sports Sciences. Likewise, the latter did not directly belong to the body of higher education and their teaching behavior was influenced by university training, in particular, by learning practical disciplines such as athletics, and gymnastics, etc. This type of training, instilled in our respondents.

We believe that educational experiences, whether they come from sports coaching or student movement coaching, and can improve specific attitudes. The accumulation of multiple specific experiences, influence the way in which the teacher apprehends his didactic intervention and the relationships that he will put in place, during the interaction with their students.

The particular distinction of the PE sector, in comparison with the general education sector, lies in the existence of the subject “practical pedagogy”. This is provided in high schools by students undergoing training at the University of Sports Sciences.

The various practical activities of the practical pedagogy internship allow the interns to acquire new experiences and also to know the role that a PE teacher plays, both in his relationship to students, to other teachers, and to various stakeholders. Who work in the educational institution. They provide opportunities to apply forms of teaching practice. Otherwise, it also allows student interns to learn the mechanisms and needs of a PE teacher in a school environment.

Ultimately, the third result we have reached therefore relates to the generational effect, and which is influenced by socialization at the university. Regarding the older generations, we can say that professional socialization has influenced their teaching practice. Indeed, the lack of continuous and in-depth training in pedagogy is reflected above all in the repetition of concrete examples. Most inspectors seem to favor technical training over scientific explanations. Even if PE is not a science but a practice, it can nevertheless be inspired by scientific results and lead to experimental or clinical research (Parlebas, 1976). All these reasons led to the formation of this pedagogical behavior.

Thus, when the teacher is effective in demonstrating his lessons, he ensures the rapid transmission of the educational content at the same time as the anchoring of certain values in the minds of the students such as respect, punctuality and ambition. When the teacher follows the evolution of scientific advances in the field of PE, he also becomes able to inculcate several skills that contribute to the success of the learner at all levels.

A good PE teacher is able to teach a variety of comprehensive skills, allowing learners to apply and intelligently select what they deem appropriate, in order to achieve their goals. This teacher is able to organize the objectives of the lesson and the pedagogical acts, in a structured way and values the mastery of execution and the way to achieve a result or performance.

Finally, the personal history of the students influences the way in which they conceive of the teaching profession. Both the family framework (primary socialization) and the school framework as well as the experiences acquired in the youth movements who live in a changing society in the sports associations (secondary socialization), bring a certain amount of knowledge, knowledge, beliefs, values which structure the personality of the students, and their conception of teaching.

It seems necessary for teachers to acquire effective verbal and non-verbal communication skills. One of the essential factors for the success or failure of students is the quality of the relationship and the way in which the teacher constructs this interaction with the students. Above all, it is certainly effective for apprentices who are weaker in human relations and communication skills.

Thus, teaching can be defined as a process planned by the teacher, to create a lasting change in the behavior of the pupils, and which takes place in the form of a cooperative relationship. The stronger this relationship and communication skills, the more profound the outcome of this interaction, and the more effective the learning will be.

On the other hand, the material environment and the mobilization of resources in a very specific way, solving the difficulties encountered by the pupils in the realization of the technical gestures, these conditions play a primordial role in the learning of the PE, in particular, and the practice teachers in their practice.

The strengths of our study lie in the choice of the studied and observed and well characterized population. We also used validated and recognized methodological tools, namely semi-structured interview and direct observation in the field to assess behaviors and attitudes, for a significant period. However, it is important to point out and take into account some limitations: the first is that this study is cross-sectional, and unlike longitudinal studies, it may seem less useful. The second limitation is frequently revealed in most research that takes a qualitative approach; this is the small number of the population studied.

To summarize, we can affirm that the socio-cultural origin, the type of training received and the generational effect play a primordial role in the pedagogical behavior of the PE teacher in a teaching setting. Likewise, social structures are reproduced within the family framework and are also part of daily practice. Thus, social roles are not data imposed on individuals, but rather social constructions that appear and are transformed at the heart of multiple interactions. Moreover, by distinguishing the different types of pedagogical practices discussed in this work, we were able to establish links between our method of approach, and the theoretical framework of socialization as it was developed in particular by Goffman (1974).

## Conclusion

The first objective of our research was to present the social characteristics of the studied population and to draw general conclusions about the social origins of the Tunisian teachers of physical education. Then we would go to a second place, spot the link between socialization, and the pedagogical practice of physical education teachers in Tunisian schools.

More precisely, we wanted to underline the influence of the socialization of teachers on their conceptions and their modes of interaction with the pupils within the framework of teaching, and on their capacity to transmit the learning outcomes. Direct observation in the field is carried out during 50 physical education and sports sessions led by PE teachers. The three results retained are the socio-cultural origin, the type of training and the generational effect.

The analysis of the different results of the didactic observation made it possible to deduce that teachers, men and women, do not apply the educational content in the same way. Indeed, we were able to observe that the contents lead to an unequal distribution of knowledge and skills in PE, due to the fact that our respondents had a popular primary socialization. Second, this differentiation can also be linked to the institutional environment, the type of school audience, and the socio-professional environment.

In addition, we can say that from a similar primary socialization, and differences exist between the ways of teaching PE at school. Indeed, during PE lessons, they are not attentive to the same behaviors, they enter into activities in a way that is specific to them and are often close to the teaching they have received but also to the types of sport that they have received they were practicing.

Good teaching practices are a fundamental issue in the teaching of PE, it is not a gift, but a skill that every teacher can develop through interaction, and learning skills. Our research results open the field to different avenues, we believe to continue our research by a survey on the socialization of research teachers in sports sciences as elite influencing the convictions, and interests of young students.

## Data Availability

The raw data supporting the conclusion of this article will be made available by the authors, without undue reservation.

## Footnotes

1 EP physical education.

## References

[B1] Arnaud P. (1992). les savoirs du corps. PUF, 112.

[B2] Becker H. (2004). “Some Ideas on Interaction,” in Alain Blanc, the art of the field Paris, 245–255.

[B3] Bourdieu P. Passeron J. C. (1970). Reproduction. The Functions of the Education System. Paris: Métailié.

[B4] Bourdieu P. Passerons J. C. (1964). The Heirs: The Students and the Culture. Paris: Midnight editions.

[B5] Bourdieu P. (1980). The Practical Sense. Paris: Midnight.

[B6] Bourdieu P. (1979). The Social Critical Distinction of Judgment. Paris: Éditions de Minuit.

[B7] Bulmer H. (1962). Society as Symbolic Interaction, Behavior and Social Processes (Boston: Houghton Mifflin), 179–192.

[B8] Cunff C. Hugon M. (2011). Interaction in the Group and Learning, 87.

[B9] Darmon M. (2006). Socialization. 2nd edition, p17.

[B10] Darmon M. (2010). Socialization. 3rd edition, p18.

[B11] Draelants H. Ballatore M. (2014). Cultural Capital and School reproductionA Critical Assessment, French Review of Pedagogy, 186.

[B12] Dubar C. (1991). Socialization: Construction of Social and Professional Identities. Paris.

[B14] Durkheim É. (19971937). The Rules of the Sociological Method. 1st edition. PUF.

[B15] Fence G. (2010). Non-verbal Communication. Paris, 98.

[B16] Feyfant A. (2008). Effect of Teaching Practices on Learning. PUF, 7.

[B17] Garfinkel H. (1967). Studies in Ethnomethodology. Englewood Cliffs, NJ: PrenticeRoom.

[B18] Goffman E. (1974). Rites of Interaction. Paris.

[B19] Guichard J. Huteau M. (2007). Guidance and Professional Integration, 119. Paris: PUF.

[B20] Huteau M. (2018). Édouard Claparède (1873-1940) et l'orientation professionnelle. Bull. de Psychol. 554, 623–631. 10.3917/bupsy.554.0623

[B21] Lautrey J. (19951980). Social Class, Family Environment, Intelligence. 1st ed. Presses Universitaires de France.

[B22] Mehrabian A. Wiener M. (1967). Decoding of Inconsistent Communications. J. Pers Soc. Psychol. 6 (1), 109–114. 10.1037/h0024532 6032751

[B29] Montousse M. Renurd G. (1999). 100 Fact Sheets for Understanding Sociology. Paris.

[B23] Parsons T. Bales R. (1955). Family, Socialization and Interaction Processes. New York: Free Press of Glencoe.

[B30] Parlebas P. (1976). Physical Activities and Motor Education. Paris: Physical and sports education.

[B28] Parlebas P. (1986). Elements of the Sociology of Sport. Paris: PUF.

[B24] Quitout M. (2007). Let's Speak Tunisian Arabic. Paris.

[B26] Streeck J. Knapp M. L. (1992). “The Interaction of Visual and Verbal Features in Human Communication,” in Advances in Non-verbal Communication. Editor Poyatos F., 3–23. 10.1075/z.60.06str

